# Reproductive Health Outcomes among Adolescent and Young Adult Cancer Patients: A Systematic Review and Meta-Analysis

**DOI:** 10.3390/cancers15061707

**Published:** 2023-03-10

**Authors:** Niki Oveisi, Vicki Cheng, Ursula Ellis, Stuart Peacock, Helen McTaggart-Cowan, Lori A. Brotto, Jonathan Loree, Gillian E. Hanley, Sharlene Gill, Meera Rayar, Amirrtha Srikanthan, Mary A. De Vera

**Affiliations:** 1 Faculty of Pharmaceutical Sciences, University of British Columbia, Vancouver, BC V6T 1Z3, Canada; 2 Collaboration for Outcomes Research and Evaluation, Vancouver, BC V6T 1Z3, Canada; 3 Woodward Library, University of British Columbia, Vancouver, BC V6T 1Z3, Canada; 4 BC Cancer, Vancouver, BC V5Z 4E6, Canada; 5 Faculty of Health Sciences, Simon Fraser University, Burnaby, BC V3T 0N1, Canada; 6 Faculty of Medicine, Vancouver, University of British Columbia, BC V6T 1Z3, Canada; 7 Division of Medical Oncology, Department of Medicine, Faculty of Medicine, University of British Columbia, Vancouver, BC V6T 1Z3, Canada; 8 Faculty of Medicine, University of Ottawa, Ottawa, ON K1N 6N5, Canada; 9 Division of Medical Oncology, Department of Medicine, The Ottawa Hospital, Ottawa, ON K1H 8L6, Canada; 10 The Ottawa Hospital Research Institute, Ottawa, ON K1H 8L6, Canada; 11 Centre for Health Evaluation and Outcome Sciences, Vancouver, BC V6Z 1Y6, Canada

**Keywords:** reproductive health, cancer survivorship, adolescent and young adult, cancer treatment

## Abstract

**Simple Summary:**

Reproductive health is an important consideration for adolescents and young adults (AYAs, aged 15–39 years) with cancer. Our systematic review and meta-analysis synthesized the current literature on the impacts of AYA cancer on reproductive health outcomes. We searched EMBASE and MEDLINE from 1 January 2000 to 26 January 2022 to capture observational studies exploring impacts of AYA cancer on reproductive health outcomes compared to controls. A total of 21 studies were included, and 62 outcomes were explored across all studies. We classified these outcomes in a sex-based framework as fetal/neonatal (n = 26), maternal (n = 11), fetal/neonatal-maternal (n = 23), and maternal-paternal (n = 2). Our analysis showed significantly higher preterm birth, gestational diabetes, and use of fertility treatment in AYA cancer patients compared to controls. Additionally, there is a higher risk of preterm birth and low APGAR score at birth for AYA cancer patients who receive radiation compared to controls. This review provides evidence of impacts of AYA cancer on reproductive health outcomes.

**Abstract:**

Background: This systematic review and meta-analysis (SRMA) aimed to synthesize the current literature on the impacts of adolescent and young adult (AYA, ages 15–39 years) cancer on reproductive health outcomes. Methods: EMBASE and Medline were searched from 1 January 2000 to 26 January 2022 for observational studies that included individuals with AYA cancer and controls which evaluated reproductive health outcomes. We used random effects models and 95% confidence intervals to obtain pooled measures of associations between AYA cancer, cancer treatment, and reproductive health outcomes. Results: The search identified 8625 articles; 21 were included. 62 reproductive outcomes were assessed and classified according to a sex-based framework as fetal/neonatal (n = 26), maternal (n = 11), fetal/neonatal-maternal (n = 23), and maternal-paternal (n = 2). Meta-analyses of crude estimates showed significant associations between AYA cancer and outcomes including preterm birth (pooled odds ratio [pOR] 1.31; 95% CI: 1.22, 1.42), gestational diabetes (pOR 1.43; 95% CI: 1.03, 1.99), and fertility treatment (pOR 2.66; 95% CI 1.71, 4.11). We also found higher odds of preterm birth (pOR 1.65; 95% CI: 1.21, 2.26) and low APGAR score at birth (pOR 2.03; 95% CI: 1.32, 3.13) among AYA cancer patients who received radiation compared to controls. Conclusions: Our SRMA quantified impacts of AYA cancers and treatments on several reproductive health outcomes.

## 1. Introduction

The incidence of cancer diagnosed among adolescents and young adults (AYAs), that is individuals between 15 and 39 years, is increasing at an alarming rate, with studies reporting a nearly 30% increase from 1973 to 2015 [1]. The impacts of cancer and its treatment on endocrine and reproductive organ function often persist long after diagnosis and treatment [2,3]. As such, these impacts can lead to long-term psycho-oncologic challenges across multiple domains including mental [4,5,6,7], psychosocial [4,5], and reproductive and sexual health [8,9]. Due to improvements in treatments translating to higher remission rates [10], reproductive health has become an important consideration among individuals diagnosed with cancer during adolescence/young adulthood as they consider family planning.

Despite the growing literature on reproductive health outcomes in AYA cancer patients—who we define as individuals across the care continuum from diagnosis to treatment to survivorship [11]—limited synthesis precludes understanding of current evidence and knowledge gaps. In 2018, Gerstl et al. conducted a systematic review and meta-analysis of 17 studies evaluating reproductive health outcomes in females diagnosed with cancer between 0 and 25 years of age [12]. Pooled analyses showed 79% of female cancer patients experienced a live birth, of which 22% were preterm births. Moreover, females who received chemotherapy alone had a pooled estimated rate of 18% of experiencing a live birth compared with 10% of females who received radiation alone. Overall, stillbirth rates were similar for cancer patients aged 0–25 (0.01%; 95% CI: 0.00, 0.002) and controls (0.01%; 95% CI: 0.006, 0.01). Low birthweight (<2500 g) was slightly higher in cancer patients aged 0–25 (10%; 95% CI: 0.09, 0.11) than controls (6%; 95% CI: 0.05, 0.07). Despite these findings, limitations related to sex (including only females) and age (individuals 0 to 25 years) of this prior systematic review limit the ability to extrapolate findings to all AYA cancer patients [13]. To expand on, comprehensively assess, and quantify the impacts of AYA cancer on reproductive health outcomes, we conducted a systematic review and meta-analysis to synthesize reproductive health outcomes evaluated in both male and females across the entire age range of AYA cancer patients (i.e., 15 to 39 years).

## 2. Materials and Methods

### 2.1. Search Methods

We conducted a systematic review and meta-analysis that adhered to the Preferred Reporting Items for Systematic Review and Meta-Analysis Protocols 2020 guidelines (PROSPERO registration number: CRD42022313343) [14]. We incorporated principles of sex- and gender-based analyses (SGBA) [15,16] throughout conduct and reporting, which accounts for the influence of sex and gender on differences in health. In collaboration with a research librarian, we developed a literature search strategy to identify peer-reviewed, published manuscripts relating to the impact of AYA cancers on reproductive health outcomes (Table 1 and Table 2). Searches were conducted in the following databases: (1) EMBASE Ovid and (2) Ovid MEDLINE(R) and Epub Ahead of Print, In-Process, In-Data-Review & Other Non-Indexed Citations, Daily and Versions. We used database-relevant terms and keywords mapping to the following concepts: (1) AYA age range (e.g., “young adult” OR “teen” OR “youth”, etc.); (2) cancer and cancer treatment (e.g., “chemotherapy” OR “radiation” OR “cancer treatment”, etc.); and (3) reproductive health outcomes (e.g., “reproductive health” OR “stillbirth” OR “preeclampsia”, etc.). Limits were added to the search to restrict results to human studies published from 1 January 2000 to 26 January 2022. Bibliographies of included studies were hand searched for additional studies that met the criteria for this review. 

### 2.2. Study Screening and Inclusion 

Search results were uploaded onto Covidence [17], where duplicates were automatically removed. The screening was completed by two reviewers (NO and MDV). In order to be eligible for inclusion, studies had to fulfill all of the following criteria: (1) used an observational study design; (2) primarily included individuals diagnosed with cancer from 15 to 39 years and a comparator (control) group of individuals without cancer (e.g., matching AYA cancer patients with controls in databases or national surveys based on sociodemographic and/or clinical factors); and (3) evaluated reproductive health outcomes (e.g., stillbirth, gestational diabetes, preterm birth, etc.). Studies that focused on pre-cancerous lesions or pregnancy-associated cancers (those diagnosed and/or treated during pregnancy) were excluded. To ensure a comprehensive capture of studies, we did not place limits on lower or upper end of age ranges but did require that the majority of study participants were between 15 to 39 years of age, which for our purposes, we defined a priori as ≥80%. No restrictions were placed on geography, language, or availability of full text. 

### 2.3. Data Extraction and Quality Assessment

We extracted information on study characteristics (publication year, country, study design, data source, sample size, and follow-up timeline) and AYA cancer exposure (definition of exposure, type of cancer, type of treatment, inclusion/exclusion criteria, age at diagnosis, subgroup analyses, age at diagnosis, and age at study). Where feasible, we pooled reported age across studies using StataSE 17 [18] (e.g., for studies that reported mean and standard deviation). Of particular relevance to our SGBA-informed approach [15] is reported information on sex (i.e., a set of biological attributes traditionally associated with sex chromosome status) and/or gender (i.e., socially and culturally constructed roles) in included studies. Specifically, we extracted information on reported sex/gender variable(s) (e.g., sex, gender, both, and neither), corresponding groups (e.g., male/female and men/women), and definitions (where relevant/provided). Key to our systematic review and meta-analysis is reproductive health outcomes, which we define as outcomes relating to conditions of male and female reproductive systems during all life stages [19]. Aside from extracting information on reproductive health outcomes assessed, we further characterized these according to who is impacted by the outcome (mother, fetus/newborn, or father) and when the outcome was assessed (before pregnancy, during pregnancy, intrauterine, delivery, and after delivery). Finally, we extracted available measures such as counts, proportions, and rates of reproductive health outcomes and measures of associations (e.g., crude and/or adjusted odds ratio and relative risk). 

Quality assessment using the Newcastle–Ottawa Scale [20] was conducted in parallel by NO and MDV, with discrepancies discussed until a consensus was reached. The following score breakdown was adapted from McPheeters et al. for cohort and case control studies [21]: (1) “Good” (possible points range: 6–8); (2) “Fair” (possible points range: 3–5); and (3) “Poor” (possible points range: 0–2). For cross-sectional studies, the following breakdown was used [21,22]: (1) “Good” (possible points range: 7–9); (2) “Fair” (possible points range: 4–6); and (3) “Poor” (possible points range: 0–3).

### 2.4. Analysis

For our meta-analyses, we computed random effects models for reproductive health outcomes that were reported by at least two studies. This was accomplished by pooling, where reported, proportions of crude events reported in each study and obtaining crude odds ratios (OR) and respective 95% confidence intervals (CI). Where possible, we conducted stratified analyses to evaluate impacts of cancer treatments. As crude events were rarely reported according to type of cancer treatment, a generic inverse-variance approach was used to obtain pooled estimates. Heterogeneity was assessed using the chi-squared test, with *p* < 0.10 indicating significant heterogeneity as opposed to *p* < 0.05, as the test is low in power when studies have small sample sizes or are few in number [23]. As an added measure, we also used the I^2^ test for inconsistency and interpreted it according to Cochrane’s recommendations with (1) 0–40% indicating little to no heterogeneity; (2) 30–60% indicating moderate heterogeneity; (3) 50–90% indicating substantial heterogeneity; and (4) ≥75% indicating considerable heterogeneity [23]. Forest plots and funnel plots were constructed for all pooled analyses. All analyses were conducted using RevMan5 [24].

## 3. Results

### 3.1. Search Results

**Figure 1:**
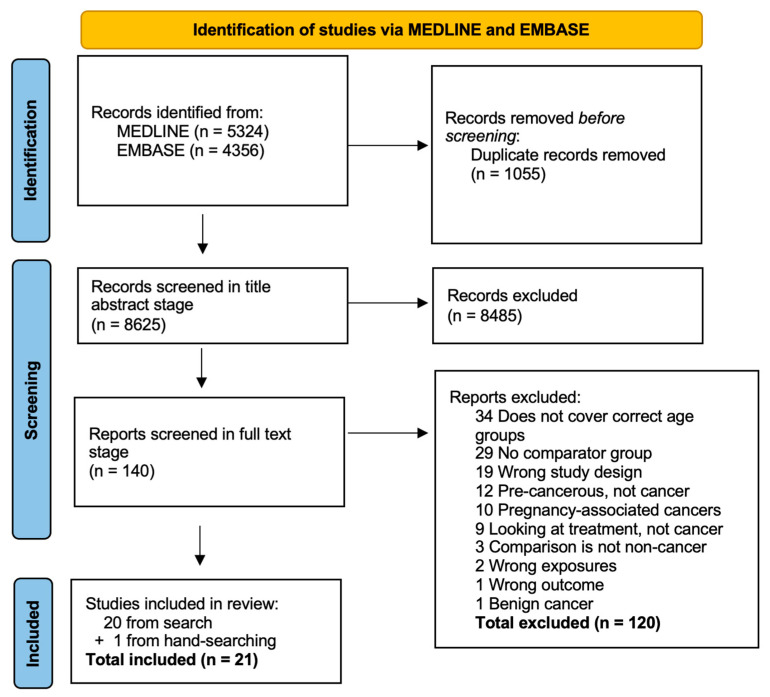
PRISMA flow diagram.

Our search strategy resulted in 8625 original citations from 1 January 2000 to 26 January 2022 (Figure 1). The main reasons for excluding 120 citations in full-text screening were: incorrect study design (n = 19); lack of a comparator group (n = 29); and participants not representative of AYA age range (n = 34). We also excluded studies that focused on pre-cancerous lesions or pregnancy-associated cancers (n = 22). Screening resulted in a total of 20 studies eligible for inclusion, and handsearching yielded one study, resulting in a total of 21 included studies. 

### 3.2. Study Characteristics

Table 3 summarizes the characteristics of the included studies. All 21 studies were conducted in high-resource countries [25]: (United States (n = 9), Canada (n = 1) [26], Taiwan (n = 2) [27,28], Norway (n = 3) [29,30,31], Finland (n = 1) [32], Denmark (n = 1) [33], Germany (n = 1) [34], South Korea (n = 1) [35], Australia (n = 1) [36], and Sweden (n = 1) [37]. The majority of included studies (n = 20) used a cohort study design, and one study used a cross-sectional design [38]. Of those using the cohort design, study follow-up ranged from 2 to 52 years. While a few studies evaluated specific types of cancer such as breast (n = 3) [34,35,39], cervical (n = 1) [40], and nasopharyngeal (n = 1) [28], the majority of studies (n = 16) did not focus on a single cancer (potentially included but not limited to: thyroid, breast, blood and leukemia, lymphoma, gynecologic [cervix, uterus, and ovary], intestines, gall bladder, pancreas, bone, soft tissue tumor of bone/fat, and/or skin). Regarding treatment information, seven studies provided information on treatment category (e.g., radiation, chemotherapy, etc.) [9,27,33,36,39,41,42], and two studies provided information on dosage and/or location of treatment [9,33]. 

Altogether, studies included a total of 102,041 AYA cancer patients. Age is an important consideration; the majority of studies (n = 18) reported age at cancer diagnosis, and all reported age at the time of the study. However, we observed variation in the reporting of age, including mean and standard deviation or proportion according to varying age categories. The pooled AYA cancer age at diagnosis was 31.42 (95% CI: 29.49, 33.36), and pooled AYA cancer age at the time of study was 32.59 (95% CI: 31.09, 34.10). The majority of studies (n = 15) studied only females. On inspection, six of these studies conflated sex and gender terminology (i.e., authors would refer to sex but use female and woman interchangeably). Six studies included both females and males, and of these, three conflated sex and gender terminology (i.e., authors would refer to sex but use male/female and men/women interchangeably). Finally, quality assessment of included studies resulted in a “Good” ranking on all cohort studies, with scores ranging from 6 to 8, and a “Poor” ranking (score = 3) on the one cross-sectional study.

### 3.3. Reproductive Health Outcomes

**Figure 2:**
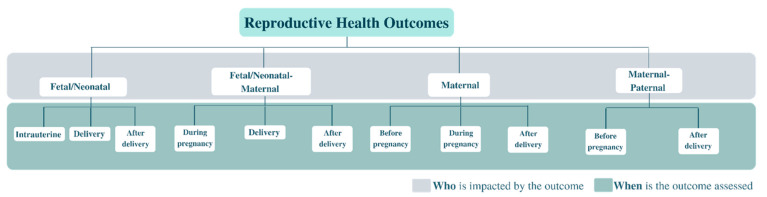
Sex-based framework of AYA cancer reproductive health outcomes based on systematic review (N = 21).

Impacts of AYA cancer were reported on a total of 62 reproductive health outcomes across the 21 included studies. As all studies reported sex as male and female, we categorized outcomes according to who is impacted and when the outcome is assessed. This led to the development of a sex-based framework for conceptualizing reproductive health outcomes as: (1) fetal/neonatal outcomes affecting the fetus or baby and assessed intrauterine, at delivery, and after delivery (n = 26); (2) maternal outcomes affecting the birth mother (with cancer) and assessed before pregnancy, during pregnancy, and after delivery (n = 11); (3) fetal/neonatal-maternal outcomes that affect both fetus/baby and birth mother and assessed during pregnancy, delivery, and after delivery (n = 23); and (4) maternal-paternal outcomes that may affect either birth mother (with cancer) or birth father (with cancer) and assessed before pregnancy and after delivery (n = 2). Figure 2 illustrates this framework, and Table 4 lists all 62 extracted outcomes, corresponding studies, and reported crude and adjusted measures of association where available.

### 3.4. Meta-Analysis

There were 17 reproductive health outcomes that were evaluated by at least two or more included studies, thereby enabling a meta-analysis to obtain pooled measures of associations.

#### 3.4.1. Fetal/Neonatal Outcomes (n = 10)

**Figure 3:**
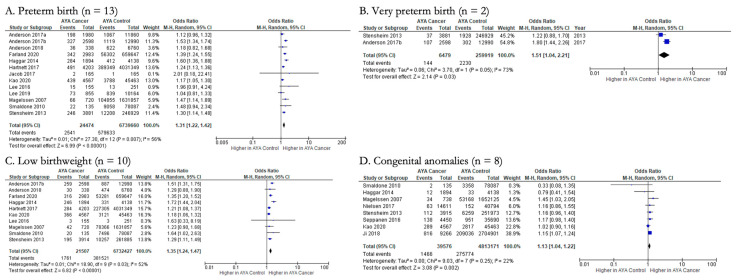
Forest plots for significant fetal/neonatal outcomes reported by two or more studies (n = 4). (**A**). [27,28,29,31,34,35,36,39,40,42,43,44,45], (**B**). [31,42], (**C**). [27,28,29,31,36,39,40,42,44,45], (**D**). [27,29,31,32,33,36,37,40].

**Figure 4:**
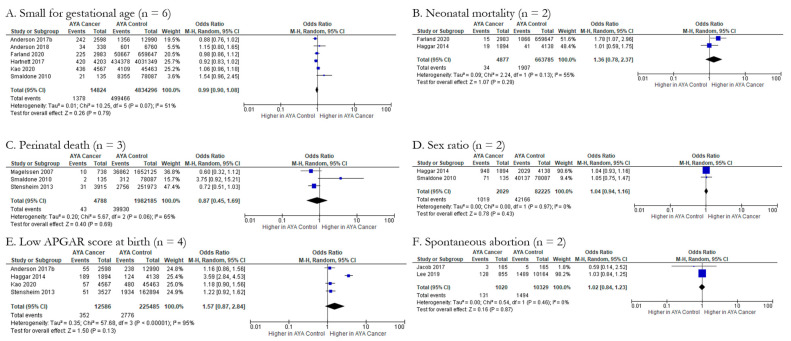
Forest plots for non-significant fetal/neonatal outcomes reported by two or more studies (n = 6). (**A**). [27,39,40,42,44,45], (**B**). [36,44], (**C**). [29,31,40], (**D**). [36,40], (**E**). [27,31,36,42], (**F**). [34,35].

There were 10 fetal/neonatal outcomes that we were able to pool (Figure 3 and Figure 4), and of these, we found that offspring of AYA cancer patients had significantly higher odds of preterm birth, very preterm birth, low birthweight, and congenital anomalies compared to controls. As the most common of these outcomes, thirteen studies examined the impact of AYA cancer (n = 24,474) on the odds of newborn preterm birth compared to AYA controls (n = 6,739,660) (Figure 3A). Pooling resulted in a pooled OR (pOR) of 1.31 (95% CI: 1.22, 1.42), indicating a significantly higher odds of newborn preterm birth for those with AYA cancer. For this outcome, there is evidence of moderate to substantial heterogeneity across studies (Chi-squared statistic: 27.30, *p* = 0.007; I^2^ = 56%). Two studies analyzed the impact of AYA cancer (n = 6479) on the likelihood of very preterm birth, which they defined as birth at <34 weeks gestational age, in comparison to AYA controls (n = 259,919), with a pOR of 1.51 (95% CI: 1.04, 2.21) (Figure 3B). There was evidence of substantial heterogeneity across studies (Chi-squared statistic: 3.70, *p* = 0.05; I^2^ = 73%). Meta-analyses also showed the association between AYA cancer and low birthweight (pOR 1.35; 95% CI: 1.24, 1.47) and congenital anomalies (pOR 1.13; 95% CI: 1.04, 1.22), respectively (Figure 3C,D).

**Figure 5:**
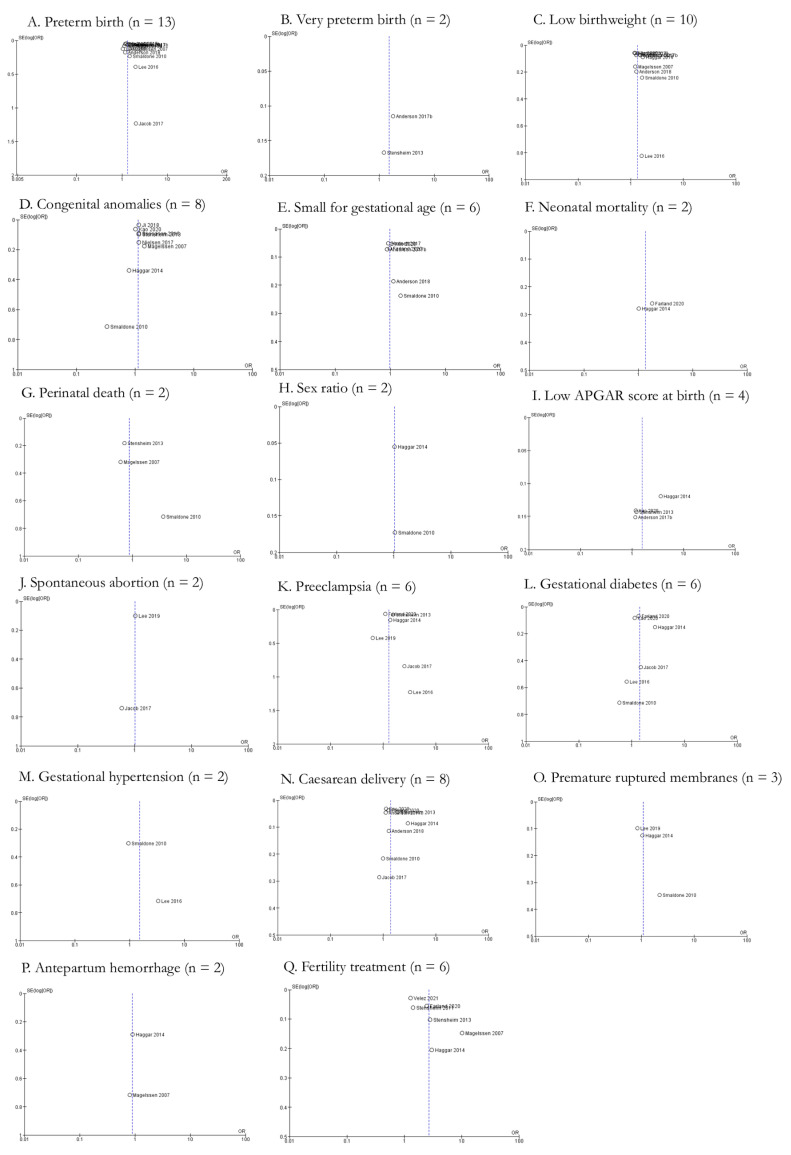
Funnel plots of meta-analyses of AYA cancer and reproductive health outcomes (n = 17). (**A**). [27,28,29,31,34,35,36,39,40,42,43,44,45], (**B**). [31,42], (**C**). [27,28,29,31,36,39,40,42,44,45], (**D**). [27,29,31,32,33,36,37,40]. (**E**). [27,39,40,42,44,45], (**F**). [36,44], (**G**). [29,31,40], (**H**). [36,40], (**I**). [27,31,36,42], (**J**). [34,35], (**K**). [28,31,34,35,36,44], (**L**). [27,28,34,36,40,44], (**M**). [28,40], (**N**). [27,31,34,36,39,40,42,44], (**O**). [35,36,40], (**P**). [29,36], (**Q**). [26,29,30,31,36,44].

However, meta-analysis also showed non-significant associations between AYA cancer and a number of fetal/neonatal outcomes including small for gestational age, neonatal mortality, perinatal death, sex ratio, low APGAR score at birth, and spontaneous abortions. Six studies explored the impact of AYA cancer (n = 14,824) on the odds of a small for gestational age newborn compared to controls (n = 4,834,296), with a pOR of 0.99 (95% CI: 0.90, 1.08) (Figure 4A). Furthermore, the meta-analysis indicated moderate to substantial heterogeneity across studies (Chi-squared statistic: 10.25, *p* = 0.07; I^2^ = 51%). Two studies evaluated neonatal mortality across a total of 4877 AYA cancer patients compared to 663,785 controls, with a resultant pOR of 1.36 (95% CI: 0.78, 2.37) (Figure 4B). The meta-analysis indicated moderate to substantial heterogeneity across the studies (Chi-squared statistic: 2.24, *p* = 0.13; I^2^ = 55%). Perinatal death (pOR 0.87; 95% CI: 0.45, 1.69), sex ratio (pOR 1.04; 95% CI: 0.94, 1.16), low APGAR score at birth (pOR 1.57; 95% CI: 0.87, 2.84), and spontaneous abortions (pOR 1.02; 95% CI: 0.84, 1.23) did not have significantly higher odds in AYA cancer patients compared to controls (Figure 4C–F). Funnel plots for all fetal/neonatal health outcomes were generated and indicate various levels of publication bias (Figure 5A–J).

#### 3.4.2. Maternal Outcomes (n = 3)

**Figure 6:**
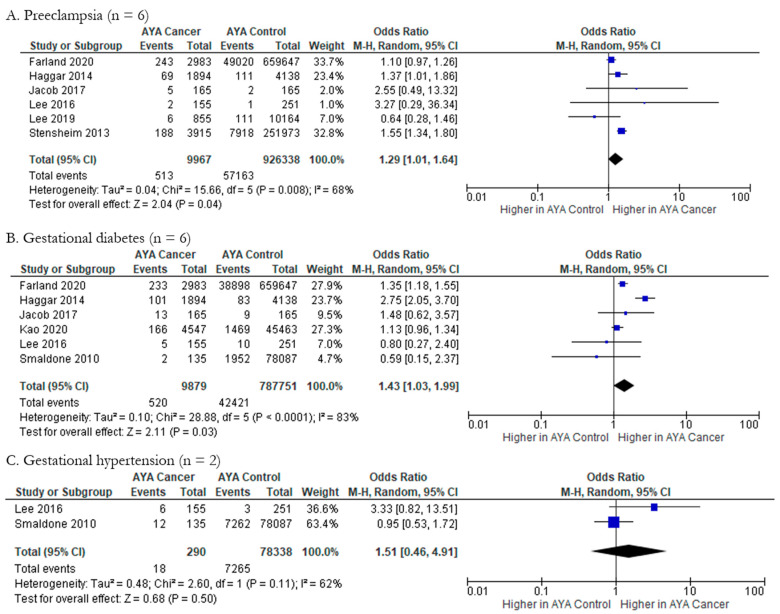
Forest plots for maternal health outcomes reported by two or more studies (n = 3). (**A**). [28,31,34,35,36,44], (**B**). [27,28,34,36,40,44], (**C**). [28,40].

Meta-analysis was feasible for three maternal outcomes, with pooled results showing that preeclampsia and gestational diabetes were significantly associated with AYA cancer diagnosis, and gestational hypertension was not. The impact of AYA cancer (n = 9967) on preeclampsia compared to those without AYA cancer (n = 926,338) was explored by six studies, with meta-analysis yielding a pOR of 1.29 (95% CI: 1.01, 1.64) (Figure 6A). There was evidence for substantial to considerable heterogeneity among studies (Chi-squared statistic: 15.66, *p* = 0.008; I^2^ = 68%). The odds of gestational diabetes in AYA cancer patients (n = 9879) compared to controls (n = 787,751) was evaluated in six studies in this review; pooling showed that there are higher odds of gestational diabetes in AYA cancer patients (pOR 1.43; 95% CI: 1.03, 1.99). (Figure 6B). Additionally, heterogeneity was substantial to considerable in this outcome (Chi-squared statistic: 28.88, *p* = <0.0001; I^2^ = 83%). Lastly, meta-analysis did not indicate higher likelihood of gestational hypertension (pOR 1.51; 95% CI 0.46, 4.91) (Figure 6C) in AYA cancer patients (n = 290) compared to controls (n = 78,338). Heterogeneity was substantial in this outcome (Chi-squared statistic: 2.60, *p* = 0.11; I^2^ = 62%). Funnel plots for all maternal health outcomes were also generated and indicate various levels of publication bias (Figure 5K–M).

#### 3.4.3. Fetal/Neonatal-Maternal Outcomes (n = 3)

**Figure 7:**
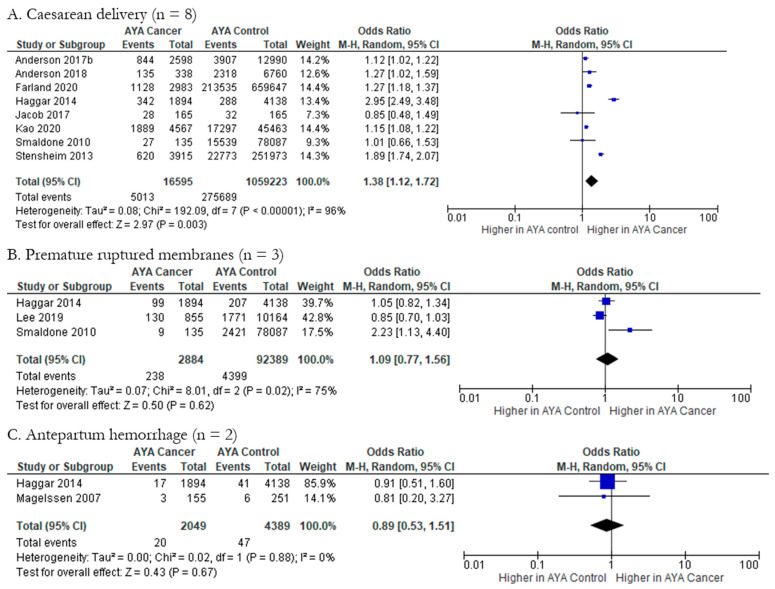
Forest plots for fetal/neonatal-maternal health outcomes reported by two or more studies (n = 3). (**A**). [27,31,34,36,39,40,42,44], (**B**). [35,36,40], (**C**). [29,36].

Meta-analyses were feasible for three fetal/neonatal-maternal outcomes, namely caesarean delivery, premature ruptured membranes, and antepartum hemorrhage. First, the likelihood of caesarean delivery in AYA cancer patients (n = 16,595) compared to controls (n = 1,059,223) was explored by eight studies (Figure 7A); pOR (1.38; 95% CI: 1.12, 1.72) indicates higher odds of caesarean delivery in AYA cancer patients compared to controls. Heterogeneity in this outcome was considerable (Chi-squared statistic: 192.09, *p* < 0.00001; I^2^ = 96%). AYA cancer patients (n = 2884) did not have higher odds of premature ruptured membranes (pOR 1.09; 95% CI 0.77, 1.56) compared to controls (n = 92,389) (Figure 7B). The meta-analysis of premature ruptured membranes showed substantial to considerable heterogeneity (Chi-squared statistic: 8.01, *p* = 0.02; I^2^ = 75%). Lastly, AYA cancer patients (n = 2049) did not have significantly higher odds of antepartum hemorrhage compared to controls (n = 4389) (pOR 0.89; 95% CI 0.53, 1.51) (Figure 7C), and heterogeneity was not present in this analysis (Chi-squared statistic: 0.02, *p* = 0.88; I^2^ = 0%). Funnel plots for all fetal/neonatal-maternal health outcomes were also generated and indicate various levels of publication bias (Figure 5N–P).

#### 3.4.4. Maternal-Paternal Outcomes (n = 1)

**Figure 8:**
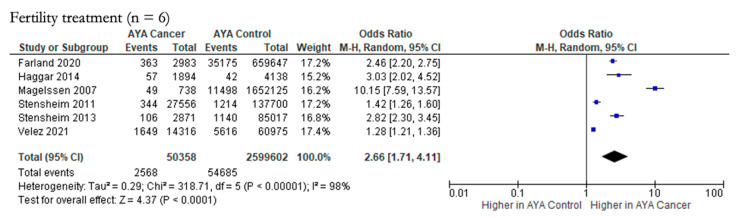
Forest plot for maternal-paternal health outcome reported by two or more studies (n = 1) [26,29,30,31,36,44].

The likelihood of requiring fertility treatments in female and male AYA cancer patients (n = 50,358) compared to controls (n = 2,599,602) was explored by six studies (Figure 8), and there was a significantly higher odds of requiring fertility treatments in male and female AYA cancer patients compared to male and female controls (pOR 2.66; 95% CI 1.71, 4.11). Heterogeneity was considerable in this outcome as well (Chi-squared statistic: 318.71, *p* < 0.00001; I^2^ = 98%). The funnel plot for this outcome indicates publication bias (Figure 5Q).

#### 3.4.5. Impact of Treatment

**Figure 9:**
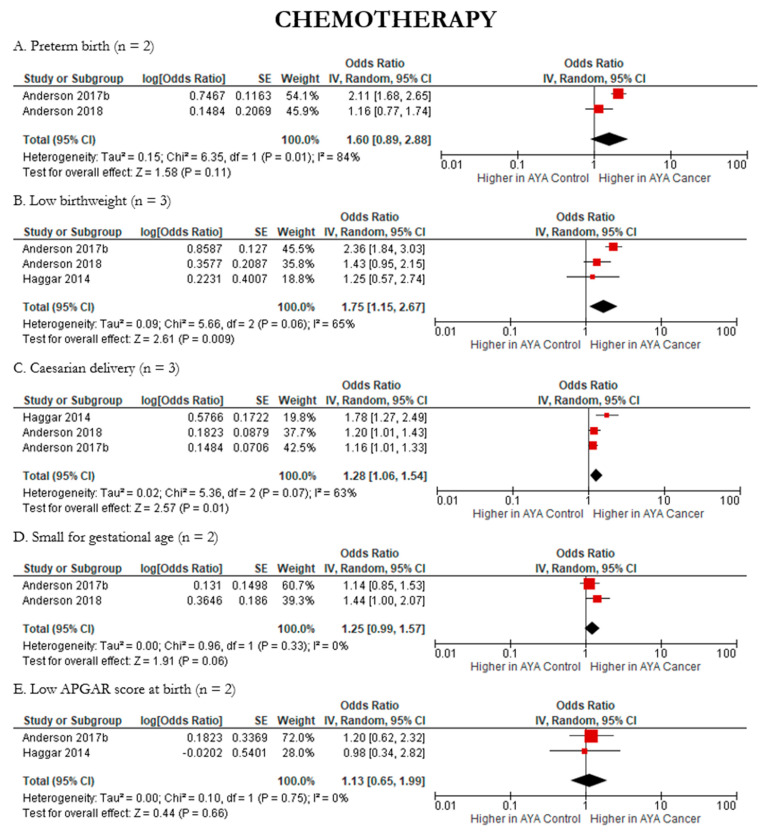
Forest plots of meta-analyses of chemotherapy and reproductive health outcomes (n = 5). (**A**). [39,42], (**B**). [36,39,42], (**C**). [36,39,42], (**D**). [39,42], (**E**). [36,42].

**Figure 10:**
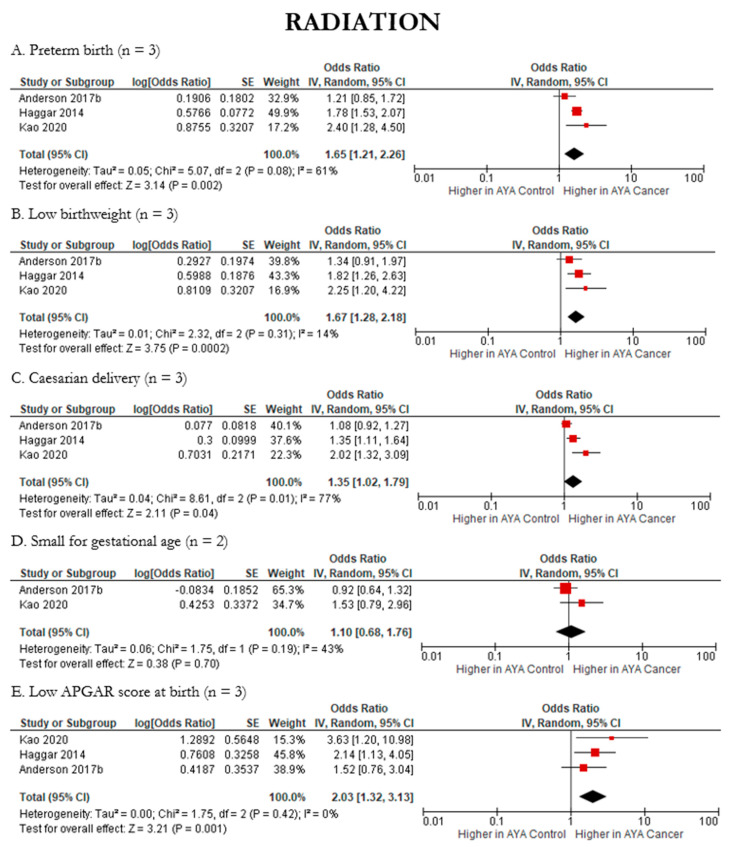
Forest plots of meta-analyses of radiation and reproductive health outcomes (n = 5). (**A**). [27,36,42], (**B**). [27,36,42], (**C**). [27,36,42], (**D**). [27,42], (**E**). [27,36,42].

**Figure 11:**
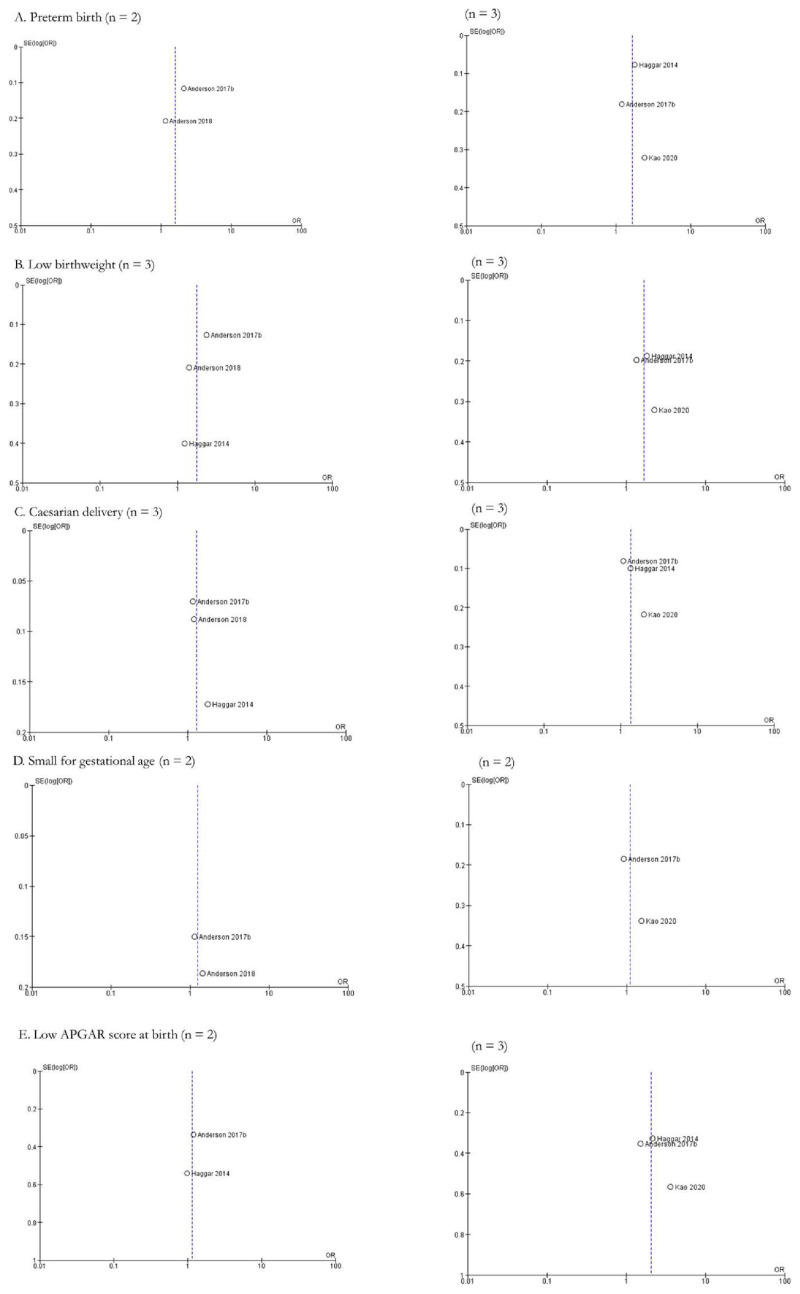
Funnel plots of meta-analyses of cancer treatment (chemotherapy or radiation compared to controls) and reproductive health outcomes (n = 5). (**A**). chemotherapy: [39,42]; radiation: [27,36,42], (**B**). chemotherapy: [36,39,42]; radiation: [27,36,42], (**C**). chemotherapy: [36,39,42]; radiation: [27,36,42], (**D**). chemotherapy: [39,42]; radiation: [27,42], (**E**). chemotherapy: [36,42]; radiation: [27,36,42].

Where feasible, we also assessed the impacts of AYA cancer treatments on the following reproductive health outcomes: low birthweight, caesarian delivery, preterm birth, low APGAR score at birth, and small for gestational age. AYA cancer patients had significantly higher odds of having a newborn with low birthweight across both chemotherapy (pOR 1.75; 95% CI: 1.15, 2.67) (Figure 9B) and radiation (pOR 1.67; 95% CI: 1.28, 2.18) (Figure 10B) compared to AYA controls. Caesarean delivery followed a similar trend, with significantly higher odds in AYA cancer patients compared to controls across both chemotherapy (pOR 1.28; 95% CI: 1.06, 1.54) (Figure 9C) and radiation therapy (pOR 1.35; 95% CI 1.02, 1.79) (Figure 10C). When considering radiation, preterm birth (pOR 1.65; 95% CI: 1.21, 2.26) (Figure 9A and Figure 10A) and low APGAR score at birth (pOR 2.03; 95% CI: 1.32, 3.13) (Figure 9E and Figure 10E) were significantly higher in AYA cancer patients compared to controls. Small for gestational age remained unchanged across treatments, with neither chemotherapy nor radiation indicating higher likelihood in AYA cancer patients compared to controls (Figure 9D and Figure 10D). Details regarding heterogeneity and publication bias can be found in Figure 9, Figure 10 and Figure 11.

## 4. Discussion

This systematic review and meta-analysis aimed to synthesize current evidence on the impact of AYA cancer on reproductive health outcomes. Altogether, we included 21 studies that reported on 62 reproductive health outcomes across 102,041 AYA cancer patients. A key contribution is the development of a sex-based framework for organizing and conceptualizing reproductive health outcomes, categorizing them into fetal/neonatal (n = 26), maternal (n = 11), fetal/neonatal-maternal (n = 23), and maternal-paternal (n = 2) outcomes. Meta-analyses that were feasible showed associations between AYA cancer and eight reproductive health outcomes: fetal/neonatal outcomes of preterm birth (pOR 1.31; 95% CI: 1.22, 1.42), very preterm birth (pOR 1.51; 95% CI: 1.04, 2.21), low birthweight (pOR 1.35, 95% CI: 1.24, 1.47), and congenital anomalies (pOR 1.13; 95% CI: 1.04, 1.22); maternal outcomes of preeclampsia (pOR 1.29; 95% CI: 1.01, 1.64) and gestational diabetes (pOR 1.43; 95% CI: 1.03, 1.99); fetal/neonatal-maternal outcome of caesarean delivery (pOR 1.38; 95% CI: 1.12, 1.72); and maternal-paternal outcome of use of fertility treatment (pOR 2.66; 95% CI 1.71, 4.11). These findings align with the current literature on the implications of cancer treatment and diagnosis on reproductive health outcomes, while providing quantitative evidence regarding the size and direction of the impact.

Given the number of reproductive health outcomes extracted (n = 62) and the variability in reporting, our developed sex-based framework for organizing and conceptualizing outcomes indicates areas research has covered, how reproductive health outcomes interrelate with each other, and gaps in the current literature. Across the entire framework, a large number of outcomes (n = 45) were reported in single studies. To investigate the impact of AYA cancer on outcomes that have been reported by single studies, we require more literature exploring these outcomes. This would facilitate pooling across studies in order to estimate the true impact of AYA cancer on the outcome. Furthermore, in our meta-analysis, we found unconfirmed associations between AYA cancer and nine outcomes, including six fetal/neonatal outcomes (small for gestational age, neonatal mortality, perinatal death, sex ratio, low APGAR score at birth, and spontaneous abortions), one maternal outcome (gestational hypertension), and two fetal/neonatal-maternal outcomes (premature ruptured membranes and antepartum hemorrhage). However, this lack of statistical significance may be driven by small sample sizes for rare outcomes across studies as well as a small number of studies in the meta-analyses, which is reflected by wide confidence intervals [46]. Therefore, there is a need for more studies that explore these outcomes. Categorization of who is impacted in this framework also identifies gaps. Particularly, although our review identified two maternal-paternal outcomes (need for fertility treatment and birth rate), we did not identify any studies that specifically evaluated the impact of male AYA cancer on paternal reproductive health outcomes. A 2021 systematic review and meta-analysis by Pizzol et al. summarized the impact of cancer treatment on ejaculatory dysfunction across all males with cancer in cross-sectional and case-control studies [47]. This review established that cancer treatment involving the lower spinal cord can impact ejaculatory function (prevalence of 6.8 to 68.7%). However, AYA cancer patients were underrepresented in the review. Considering the large psychosocial and economic impact of these outcomes for male AYA cancer patients [48], there is a need for more cohort studies to adequately assess these impacts compared to control populations.

Key findings of our meta-analysis are pooled estimates that quantify the association between AYA cancer and four fetal/neonatal outcomes (preterm birth, very preterm birth, low birthweight, and congenital anomalies), two maternal outcomes (preeclampsia and gestational diabetes), one fetal/neonatal-maternal outcome (caesarean delivery), and one maternal-paternal outcome (fertility treatment). Furthermore, when we further evaluated cancer treatment, we found associations between radiation exposure and preterm birth, low birthweight, caesarian delivery, and low APGAR score at birth. Chemotherapy was associated with low birthweight and caesarian delivery. Previous research has shown that radiation has a significant impact on fertility, especially when damage occurs to the pelvic or cranial regions [49,50]. Our findings align with previous research, as our data extraction found a higher risk of premature ovarian failure (OR 3.12; 95% CI: 1.70, 5.72) [9], and pooling use of fertility treatment indicated a higher risk (pOR 2.66; 95% CI 1.71, 4.11) in AYA cancer patients. Treatment to the pelvic region can result in damage to germ cells, while treatment to the cranial regions can alter the production of sex hormones from the hypothalamic–pituitary axis. These can result in issues with fertility, such as premature ovarian failure and increased usage of fertility treatment in AYA cancer patients. However, the majority of included studies did not report reproductive health outcomes according to treatment type, and as such, we were limited in our meta-analysis of the impact of cancer treatment. Additionally, further research is needed in comparing different types of radiation and/or chemotherapy, types of cancer, the impact of dosage, as well as location of treatment on reproductive health outcomes. Nonetheless, in quantifying associations between AYA cancer and, where feasible, cancer treatment and reproductive health outcomes according to our framework, our review provides empirical evidence to guide reproductive health care and decision making for both providers and patients. Indeed, our review supports the need for oncofertility counselling both prior to and after receiving treatment for AYA cancer patients. As treatment types can impact reproductive health outcomes, this should be taken into consideration during treatment plan development. This is reflected in an included study in our review by Medica et al. [38], who found a significantly higher use of emergency contraception in AYA cancer patients (OR 2.09; 95% CI: 1.82, 1.39). A higher frequency of emergency contraception usage suggests a need for more contraceptive counselling in AYA cancer patients. Therefore, discussions regarding family planning and the potential risks of adverse reproductive health outcomes across the developed framework is warranted. This will allow AYA cancer patients to make informed decisions regarding their reproductive health and family planning, which may reduce the anxiety associated with this process after cancer. In addition to oncofertility counselling, there is a need for closer obstetrical follow-up during pregnancy and delivery. Our review provides evidence of higher risk of gestational diabetes and preeclampsia, which negatively impact pregnancy. There is also a higher risk of fetal reproductive health outcomes such as preterm birth, very preterm birth, and low birthweight, which require management both before and after delivery. By providing closer follow-up, both the pregnant person and the fetus can receive care efficiently.

Given the inquiry into reproductive health, an important consideration in our systematic review is sex (a set of biological attributes traditionally associated to sex chromosome status) and gender (socially and culturally constructed roles). All of the included studies in our review reported sex in a binary fashion (i.e., male and female) and did not report gender or sexual orientation as a sociodemographic factor. This may be a limitation of data sources used, as the majority of the included studies relied on administrative health data, where gender-diverse data are not collected. As development of our framework for organizing and conceptualizing reproductive health outcomes was informed by studies included in the systematic review, it is important to note that it is sex-based. Indeed, it is also important that future research on AYA cancer and reproductive health is guided on principles of SGBA [15]. Among studies included in our systematic review, we noted instances of conflation of sex and gender when referring to sex. For example, the term “woman” was used when referring to the sex variable collected in a database, hence conflating sex and gender. Representation and inclusion of gender is integral to the external validity of research as well as the safety and care of the target population. Research has shown that trans and non-binary folks experience significant health disparities due to lack of access to appropriate care, financial barriers, and minimal cultural competency from healthcare providers [51]. Similar trends are seen for those of non-heterosexual status [52], who are at a higher risk for certain cancers in adolescence and young adulthood [53]. Therefore, data regarding the impact of AYA cancer on reproductive outcomes stratified according to sex, gender, and sexual orientation is imperative to measure the unique impact of these factors. Although data limitations may preclude the ability to incorporate SGBA [54] when evaluating the impact of AYA cancer and treatments on reproductive health, it is important to be aware of the intersectionality between sex, gender, and sexual identities and the potential impacts on outcomes. Future studies should intentionally include valid measures of sex, gender, and sexual orientation given that each of these have known, and potentially distinct, impacts on sexual and reproductive health measures.

Strengths and limitations of our work warrant discussion. Our search strategy was developed in collaboration with a research librarian. We applied a systematic approach to categorizing reported reproductive health outcomes, resulting in the aforementioned conceptual framework that guided our meta-analysis. It is important to comment on the heterogeneity we observed across meta-analyses that we were able to conduct. The heterogeneity in our meta-analyses ranged from 0% to 98%. Given our focus on observational epidemiologic studies, this was anticipated [23]. Heterogeneity is largely explored as methodological and clinical differences in how studies were executed. In terms of methodological heterogeneity, 20 of the included studies were cohort studies, and one was a cross-sectional study. All studies were conducted in high-resource countries and utilized a form of administrative health data. However, there was large diversity in sample sizes, which can be a driver of heterogeneity. Additionally, we expect a large degree of clinical heterogeneity, as participants of observational studies are not randomized to reduce confounding and selection bias, nor is there consistency across the included studies in terms of diversity of participants, interventions, or outcomes measured [23]. In order to further explore the source of heterogeneity, it is recommended to conduct subgroup analyses and stratify by the study feature in question [23]. This was not feasible in our review, as the included studies did not report their results according to characteristics of potential heterogeneity (e.g., intervention, type of cancer, clinical setting, sociodemographic factors, etc.). Furthermore, sensitivity analyses (that is, excluding individual studies and observing the impact on heterogeneity) were not feasible, as many of our outcomes were reported by a small number of studies. Removal of studies would have resulted in even wider confidence intervals and lower accuracy of our results [23].

## 5. Conclusions

Altogether, our systematic review and meta-analysis provide a comprehensive synthesis of reproductive health outcomes among AYA cancer patients. Findings have implications for supporting the need for oncofertility counseling before and after treatment so patients and their families can make informed decisions. Guidelines for specific obstetrical follow-up during and after pregnancy is also warranted to address the entire continuum before, during, and after pregnancy, which are impacted by AYA cancer status. This review also informs future research to address reproductive health for AYA cancer patients.

## Figures and Tables

**Table 1:** EMBASE Ovid Search (1974 to 26 January 2022).

Search Line	Search Term	Hits
1	Young adult/	442,421
2	Adolescent/	1,642,976
3	(young adult* or teen* or adolescen* or youth*).ti,ab,kw.	609,445
4	1 or 2 or 3	2,127,993
5	exp Neoplasm/or exp Cancer Radiotherapy/or exp Antineoplastic agent/ or exp Early cancer diagnosis/or exp cancer chemotherapy/	6,205,318
6	(Chemotherap* or “cancer treatment*” or radiation or brachytherap* or “antineoplastic agent*” or “antitumor* drug*” or “antitumor* agent*” or antineoplastics* or “anticancer* agent**” or “anticancer* drug*” or “early detection of cancer” or “oncolog* surger*”).ti,ab,kw.	1,297,180
7	5 or 6	6,527,287
8	exp reproductive health/or exp spontaneous abortion/or exp stillbirth/or exp birth weight/ or exp small for date infant/or exp prematurity/ or exp obstetric delivery/or exp cesarean section/or exp forceps delivery/or exp vacuum extraction/or exp pregnancy diabetes mellitus/or exp maternal hypertension/ or exp “eclampsia and preeclampsia”/or exp preeclampsia/or exp HELLP syndrome/ or exp postnatal depression/or exp labor complication/or exp perinatal death/or exp perinatal mortality/ or exp fetus death/or exp pregnancy complication/or drug induced malformation/or radiation induced malformation/	603,279
9	(“Reproductive health outcome*” or “pregnancy loss*” or miscarriage* or “spontaneous abortion*” or stillbirth* or “still birth*” or “fetal death*” or “Perinatal death*” or “low birth weight*” or “low birthweight*” or “neonatal underweight” or “small for gestational age*” or “premature birth*” or “preterm birth*” or prematur* or pre-matur* or “pre-term birth*” or “pre-mature birth*” or C-section* or “cesarean section*” or “vaginal deliver*” or “forceps deliver*” or “vacuum extraction*” or “natural deliver*” or “gestational hypertens*” or “pregnancy-induced hypertens*” or “pregnancy transient hypertens*” or “pregnancy-induced diabetes” or “gestational diabetes” or “pre eclampsia” or “preeclampsia” or “pregnancy toxemia” or “pre-eclampsia” or “HELLP syndrome” or “hemolysis elevated liver enzymes and low platelets syndrome” or “postnatal depression” or “postpartum depression” or “postpartum anxiety” or “postnatal anxiety” or “pregnancy anxiety” or “pregnancy depression” or “perinatal anxiety” or “perinatal depression” or “pregnancy complication” or “Congenital Abnormalit*” or “Congenital malformation*” or “Congenital Defect*” or “Fetal Malformation*” or “Fetal Anomal*” or “Birth defect*” or “Congenital anomal*” or “Development anomal*” or “Obstetric Labor Complication*” or “Labor Complication*”).ti,ab,kw.	537,559
10	8 or 9	844,485
11	4 and 7 and 10	7022
12	limit 11 to yr = “2000–Current”	6226
13	limit 12 to “humans only (removes records about animals)”	6165
14	limit 13 to embase	4356

**Table 2:** Ovid MEDLINE(R) and Epub Ahead of Print, In-Process, In-Data-Review & Other Non-Indexed Citations, Daily and Versions(R) Search <1946 to 26 January 2022>.

Search Line	Search Term	Hits
1	Young adult/	975,082
2	Adolescent/	2,154,258
3	(young adult* or teen* or adolescen* or youth*).ti,ab,kw.	486,342
4	1 or 2 or 3	2,751,081
5	exp Neoplasms/or exp Radiotherapy/or exp Antineoplastic agents/ or exp “Early Detection of Cancer”/	4,271,519
6	(Chemotherap* or “cancer treatment*” or radiation or brachytherap* or “antineoplastic agent*” or “antitumor* drug*” or “antitumor* agent*” or antineoplastics* or “anticancer* agent**” or “anticancer* drug*” or “early detection of cancer” or “oncolog* surger*”).ti,ab,kw.	905,198
7	5 or 6	4,604,154
8	exp Reproductive Health/or exp Spontaneous Abortion/or exp Stillbirth/or exp Birth Weight/or exp Infant, Small for Gestational Age/or exp Premature Birth/or exp Premature Infant/or exp Infant, Extremely Premature/or exp Delivery, Obstetric/or exp Cesarean Section/or exp Extraction, Obstetrical/or exp Vacuum extraction, Obstetrical/or exp Obstetrical Forceps/or exp Diabetes, Gestational/ or exp Hypertension, pregnancy-induced/or exp Pre-Eclampsia/or exp HELLP Syndrome/or exp Depression, post-partum/or exp Obstetric Labor Complications/or exp perinatal death/or exp pregnancy complications/or abnormalities, drug-induced/or abnormalities, radiation-induced/	586,641
9	(“Reproductive health outcome*” or “pregnancy loss*” or miscarriage* or “spontaneous abortion*” or stillbirth* or “still birth*” or “fetal death*” or “Perinatal death*” or “low birth weight*” or “low birthweight*” or “neonatal underweight” or “small for gestational age*” or “premature birth*” or “preterm birth*” or prematur* or pre-matur* or “pre-term birth*” or “pre-mature birth*” or C-section* or “cesarean section*” or “vaginal deliver*” or “forceps deliver*” or “vacuum extraction*” or “natural deliver*” or “gestational hypertens*” or “pregnancy-induced hypertens*” or “pregnancy transient hypertens*” or “pregnancy-induced diabetes” or “gestational diabetes” or “pre eclampsia” or “preeclampsia” or “pregnancy toxemia” or “pre-eclampsia” or “HELLP syndrome” or “hemolysis elevated liver enzymes and low platelets syndrome” or “postnatal depression” or “postpartum depression” or “postpartum anxiety” or “postnatal anxiety” or “pregnancy anxiety” or “pregnancy depression” or “perinatal anxiety” or “perinatal depression” or “pregnancy complication” or “Congenital Abnormalit*” or “Congenital malformation*” or “Congenital Defect*” or “Fetal Malformation*” or “Fetal Anomal*” or “Birth defect*” or “Congenital anomal*” or “Development anomal*” or “Obstetric Labor Complication*” or “Labor Complication*”).ti,ab,kw.	399,110
10	8 or 9	798,565
11	4 and 7 and 10	8746
12	limit 11 to yr = “2000–Current”	5382
13	limit 12 to “humans only (removes records about animals)”	5324

**Table 3:** Characteristics of included studies on reproductive health outcomes among adolescent and young adult (AYA) cancer patients (N = 21).

Study	Country	Study Design	Follow-Up Timeline (Years)	Sex (% Female)	Data Cancer ^a^	Type of Cancer ^b^	AYA N	AYA Age at Diagnosis (yr)	AYA Age at Study (yr)	Quality Assessment ^c^
Anderson 2017a [43]	United States	Cohort	14	100	North Carolina Central Cancer Registry	Any	1980	Average years between diagnosis and birth = 3.5 ± 2.4	31.2 ± 5.3	7 = Good
Anderson 2017b [42]	United States	Cohort	14	100	North Carolina Central Cancer Registry	Any	2598	28.1 ± 5.5	31.1 ± 5.3	8 = Good
Anderson 2018 [39]	United States	Cohort	14	100	North Carolina Central Cancer Registry	Breast cancer	338	35 ± 3.7	35.1 ± 4.3	8 = Good
Chao 2020 [9]	United States	Cohort	2	65	Kaiser Permanente Southern California SEER ^d^ affiliated cancer registry	Any	6778	31.3 ± 6.5	Age: Number of participants (%): 15–19: 521 (7.7%) 20–29: 1706 (25.2%) 30–39: 4551 (67.1%)	8 = Good
Farland 2020 [44]	United States	Cohort	9	100	Massachusetts Cancer Registry	Any	2983	Age: Number of participants (%) = <15: 54 (2.2%) 15–26: 802 (33.1%) >26: 1566 (64.7%)	33.6 ± 5.2	7 = Good
Haggar 2014 [36]	Australia	Cohort	25	100	Western Australian Data Linkage System	Any	1894	Age: Number of participants (%) = 15–19: 739 (39%) 20–29: 98 (52%) 30–39: 170 (9%)	Age: Number of participants (%): 15–19: 193 (10%) 20–29: 841 (44%) 30–34: 550 (29%) ≥35: 310 (16%)	8 = Good
Hartnett 2017 [45]	United States	Cohort	Georgia: 18 North Carolina: 14 Tennessee: 9	100	Cancer registries in the states of Georgia, North Carolina, and Tennessee	Any	4203	Between 20–45	Age: Number of participants (%): 20–24: 250 (5.9%) 25–29: 1084 (26%) 30–34: 1479 (35%) 35–39: 1091 (26%) 40–45: 299 (7.1%)	8 = Good
Hartnett 2018 [41]	United States	Cohort	Georgia: 18 North Carolina: 14 Tennessee: 9	100	Cancer registries in the states of Georgia, North Carolina, and Tennessee. Subset of participants from Furthering Understanding of Cancer, Health, and Survivorship in Adult (FUCHSIA) Women’s Study	Any	4203	Age: Number of participants (%) = 20–24: 910 (22%) 25–29: 1412 (34%) 30–34: 1283 (31%) 35–39: 532 (13%) 40–45: 66 (2%)	Age: Number of participants (%): 20–24: 251 (6%) 25–29: 1084 (26%) 30–34: 1480 (35%) 35–39: 1089 (26%) 40–45: 299 (7%)	8 = Good
Jacob 2017 [34]	Germany	Cohort	14	100	Disease Analyzer database (IMS Health)	Breast cancer	165	Interval between breast cancer diagnosis and first pregnancy was 18 months, with a minimum of 6 months and a maximum of 10 yrs	34.6 ± 5.2	7 = Good
Ji 2018 [37]	Sweden	Cohort	52	100	Swedish Cancer Registry	Any	9266	Not reported	Median (range): 33 (16–46)	6 = Good
Kao 2020 [27]	Taiwan	Cohort	10	100	Taiwan Birth Reporting System and National Health Insurance database	Any	3531	Median: 27.1	Age: Number of participants (%): 15–24: 148 (3.3) 25–34: 28820 (63.4) ≥35 (max. 48): 1517 (33.4)	8 = Good
Lee 2016 [28]	Taiwan	Cohort	12	100	Taiwan National Health Insurance Research database	Nasopharyngeal carcinoma	155	Not reported	Age: Number of participants/overall: 15–24: 21/155 25–34: 95/155 35–44: 37/155 ≥45: 2/155	8 = Good
Lee 2019 [35]	South Korea	Cohort	6	100	National Health Information Database from the Korean National Health Insurance Service	Breast cancer	855	34.9 ± 3.8	Age: Number of participants (%): 20–29: 745 (87.1) 30–39: 110 (12.9)	8 = Good
Magelssen 2007 [29]	Norway	Cohort	Substudy 1: 11 Substudy 2: 37	38	Cancer Registry in Norway	Any	747	Substudy 1: Male: 22 (15–30) Female: 22 (15–31) Substudy 2: Group 1: Male: 25 (15–35) Female: 24 (15–35) Group 2: Male: 29 (21–35) Female: 28 (19–36)	Male: 27 (17–36) Female: 25 (17–35)	6 = Good
Medica 2018 [38]	United States	Cross-sectional	Not applicable	100	Reproductive Window Study and National Survey of Family Growth (2006–2010)	Any	616	Mean (SD) years since cancer diagnosis: 7.5 ± 5.3	Age: Number of participants (%): 18–24: 35 (5.8%) 25–30: 138 (23%) 31–35: 215 (35.8%) 36–40: 213 (35.4%)	3 = Poor
Nielsen 2017 [33]	Denmark	Cohort	34	44	Danish Cancer Registry	Any	8945	Age (%): <35: 80.3% ≥35: 19.7%	Not reported	8 = Good
Seppanen 2016 [32]	Finland	Cohort	51	49	Finnish Cancer Registry	Any	6862	0–34	Age: Number of participants (%) <20: 718 (5.1%) 20–24: 3604 (25.4%) 25–29: 5221 (36.7%) 30–34: 3389 (23.8%) 35+: 1275 (9%)	8 = Good
Smaldone 2010 [40]	United States	Cohort	17	100	University of Pittsburgh Medical Center Network Cancer Registry	Cervical	135	Not reported	Age: Number of participants (%) <24: 17725 (22.7%) 25–29: 19834 (25.4%) 30–34: 24831 (31.8%) ≥35: 15617 (20%)	7 = Good
Stensheim 2011 [30]	Norway	Cohort	37	58	Cancer Registry of Norway	Any	27556	Median: Male: 32 Female: 36	Median observation (range): Male: 6.2 (0–29.8) Female: 5.0 (0–29.8)	8 = Good
Stensheim 2013 [31]	Norway	Cohort	37	47	Cancer Registry of Norway	Any	3915	Female: Nulliparous: 24.0 ± 5.1 Primiparous: 27.3 ± 4.5	Female: Nulliparous: 29.1 ± 4.9 Primiparous: 31.1 ± 4.4	8 = Good
								Male: Nulliparous: 25.1 ± 5.0 Primiparous: 28.9 ± 5.0	Male: Nulliparous: 30.7 ± 4.9 Primiparous: 32.7 ± 5.0	
Velez 2021 [26]	Canada	Cohort	9	100	Ontario Cancer Registry	Any	14316	31.4 ± 6.3	Median follow-up time (SD): 13.1 ± 0.08	8 = Good

^a^ Cancer registry often linked to other databases for sociodemographic and/or perinatal information. ^b^ “Any” cancer includes but is not limited to: thyroid, breast, blood and leukemia, lymphoma, gynecologic (cervix, uterus, and ovary), intestines, gall bladder, pancreas, bone, soft tissue tumor of bone/fat, and/or skin. ^c^ The Newcastle–Ottawa Scale for assessing the quality of non-randomized studies. ^d^ The Surveillance, Epidemiology, and End Results (SEER) Program provides information on cancer statistics in the United States.

**Table 4:** Summary of outcomes and their measures of association from included studies (N = 62).

Outcome	Study	Crude Event Rates Reported? (Y/N)	Crude Estimate (95% Confidence Interval)	Adjusted Estimate (95% Confidence Interval)
**MATERNAL HEALTH OUTCOMES (n = 11 outcomes)**				
Before pregnancy (n = 3 outcomes)				
Emergency contraception use				
1	Medica 2018 [38]	Y	OR 2.09 (1.82, 1.39)	- ^b^
Known or suspected abnormality of pelvic organs				
1	Jacob 2017 [34]	Y	OR 1.00 (0.41, 2.47)	-
Premature ovarian failure				-
1	Chao 2020 [9]	Y	OR 3.12 (1.70, 5.72) ^a^	IRR 2.87 (1.56, 5.28)
During pregnancy (n = 4 outcomes)				
Preeclampsia				
1	Farland 2020 [44]	Y	RR 1.08 (0.95, 1.22)	RR 1.05 (0.92, 1.19)
2	Haggar 2014 [36]	Y	OR 1.37 (1.01, 1.86) ^a^	RR 1.44 (1.13, 1.87)
3	Jacob 2017 [34]	Y	OR 2.54 (0.49, 13.32)	-
4	Lee 2016 [28]	Y	OR 3.27 (0.29, 36.3)	OR 3.48 (0.31, 39.1)
5	Lee 2019 [35]	Y	OR 0.64 (0.28, 1.46) ^a^	OR 0.61 (0.27, 1.40)
6	Stensheim 2013 [31]	Y	OR 1.55 (1.34, 1.80) ^a^	-
Gestational diabetes				
1	Farland 2020 [44]	Y	RR 1.29 (1.13, 1.48)	RR 1.08 (0.94, 1.23)
2	Haggar 2014 [36]	Y	OR 2.75 (2.05, 3.70) ^a^	RR 1.38 (1.09, 2.98)
3	Jacob 2017 [34]	Y	OR 1.48 (0.61, 3.57)	-
4	Kao 2020 [27]	Y	OR 1.13 (0.96, 1.34) ^a^	-
5	Lee 2016 [28]	Y	OR 0.80 (0.27, 2.40)	OR 0.79 (0.26, 2.40)
6	Smaldone 2010 [40]	Y	RR 0.61 (0.15, 2.46)	-
Gestational hypertension				
1	Lee 2016 [28]	Y	OR 3.33 (0.82, 13.5)	OR 3.30 (0.79, 13.8)
2	Smaldone 2010 [40]	Y	RR 0.95 (0.52, 1.72)	-
Maternal anemia				
1	Haggar 2014 [36]	Y	OR 1.18 (0.69, 2.00) ^a^	RR 1.31 (0.71, 2.19)
After delivery (n = 4 outcomes)				
Postpartum hemorrhage				
1	Haggar 2014 [36]	Y	OR 1.05 (0.81, 1.34) ^a^	RR 1.08 (0.82, 1.56)
Retained placenta				
1	Haggar 2014 [36]	Y	OR 0.97 (0.71, 1.33) ^a^	RR 0.98 (0.73, 1.34)
Postpartum length of stay >5 days				
1	Haggar 2014 [36]	Y	OR 2.85 (2.33, 3.48)	RR 3.01 (1.72, 5.58)
Genito-urinary tract infections				
1	Jacob 2017 [34]	Y	OR 0.53 (0.19, 1.46)	-
**FETAL/NEONATAL HEALTH OUTCOMES (n = 26 outcomes)**				
Intrauterine (n = 3 outcomes)				
Intrauterine growth restriction				
1	Haggar 2014 [36]	Y	OR 2.88 (2.19, 3.80)	RR 1.21 (0.97, 2.06)
Intrauterine death				
1	Haggar 2014 [36]	Y	OR 1.03 (0.70, 1.51) ^a^	RR 1.07 (0.86, 1.65)
Suspected poor fetal growth				
1	Jacob 2017 [34]	Y	OR 2.11 (0.88–5.07)	-
Delivery (n = 2 outcomes)				
Low APGAR score at birth				
1	Anderson 2017b [42]	Y	OR 1.16 (0.86, 1.56) ^a^	PR 1.18 (0.87, 1.61)
2	Haggar 2014 [36]	Y	OR 3.59 (2.84, 4.53)	RR 2.83 (2.28, 3.56)
3	Hartnett 2017 [45]	N	Could not pool	-
4	Kao 2020 [27]	Y	OR 1.19 (0.90, 1.57)	OR 1.14 (0.86, 1.51)
5	Stensheim 2013 [31]	Y	OR 1.22 (0.92, 1.62) ^a^	-
Resuscitation				
1	Haggar 2014 [36]	Y	OR 1.83 (1.48, 2.26)	RR 1.66 (1.27, 2.19)
After delivery (n = 21 outcomes)				
Preterm birth				
1	Anderson 2017a [43]	Y	RR 1.17 (1.01, 1.35)	RR 1.24 (1.07, 1.43)
2	Anderson 2017b [42]	Y	OR 1.53 (1.34, 1.74) ^a^	PR 1.52 (1.34, 1.71)
3	Anderson 2018 [39]	Y	OR 1.18 (0.82, 1.68) ^a^	PR 1.10 (0.78, 1.54)
4	Farland 2020 [44]	Y	RR 1.30 (1.16, 1.46)	RR 1.19 (1.07, 1.32)
5	Haggar 2014 [36]	Y	OR 1.60 (1.36, 1.88) ^a^	RR 1.68 (1.21, 2.08)
6	Hartnett 2017 [45]	Y	OR 1.24 (1.13, 1.36) ^a^	-
7	Hartnett 2018 [41]	N	Could not pool	-
8	Jacob 2017 [34]	Y	OR 2.01 (0.18, 22.41)	-
9	Kao 2020 [27]	Y	OR 1.16 (1.04, 1.29)	OR 1.12 (1.00, 1.25)
10	Lee 2016 [28]	Y	OR 1.96 (0.91, 4.24)	OR 2.03 (0.92, 4.45)
11	Lee 2019 [35]	Y	OR 1.04 (0.81, 1.33) ^a^	OR 1.02 (0.80, 1.31)
12	Magelssen 2007 [29]	Y	OR 1.47 (1.14, 1.89) ^a^	-
13	Smaldone 2010 [40]	Y	RR 1.48 (0.94, 2.34)	-
14	Stensheim 2013 [31]	Y	OR 1.30 (1.14, 1.48) ^a^	-
Low birthweight				
1	Anderson 2017b [42]	Y	OR 1.51 (1.31, 1.75) ^a^	PR 1.59 (1.38, 1.83)
2	Anderson 2018 [39]	Y	OR 1.29 (0.88, 1.90) ^a^	PR 1.11 (0.77, 1.61)
3	Farland 2020 [44]	Y	RR 1.27 (1.13, 1.43)	RR 1.19 (1.071.32)
4	Haggar 2014 [36]	Y	OR 1.72 (1.44, 2.04) ^a^	RR 1.51 (1.23, 2.12)
5	Hartnett 2017 [45]	Y	OR 1.21 (1.08, 1.37) ^a^	-
6	Hartnett 2018 [41]	N	Could not pool	-
7	Kao 2020 [27]	Y	OR 1.19 (1.06, 1.34)	OR 1.15 (1.02, 1.30)
8	Lee 2016 [28]	Y	OR 1.63 (0.33, 8.19)	OR 1.71 (0.33, 8.89)
9	Magelssen 2007 [29]	Y	OR 1.23 (0.90, 1.68) ^a^	-
10	Smaldone 2010 [40]	Y	RR 1.65 (1.03, 2.65)	-
11	Stensheim 2013 [31]	Y	OR 1.29 (1.11, 1.49) ^a^	-
Small for gestational age				
1	Anderson 2017b [42]	Y	OR 0.88 (0.76, 1.02)	PR 0.97 (0.85, 1.11)
2	Anderson 2018 [39]	Y	OR 1.15 (0.80, 1.65) ^a^	PR 1.02 (0.72, 1.45)
3	Farland 2020 [44]	Y	RR 0.97 (0.85, 1.10)	RR 1.02 (0.89, 1.16)
4	Hartnett 2017 [45]	Y	OR 0.92 (0.83, 1.02) ^a^	-
5	Hartnett 2018 [41]	N	Could not pool	-
6	Kao 2020 [27]	Y	OR 1.08 (0.97, 1.20)	OR 1.07 (0.96, 1.19)
7	Smaldone 2010 [40]	Y	RR 1.54 (1.00, 2.46)	-
Congenital anomalies				
1	Haggar 2014 [36]	Y	OR 0.79 (0.41, 1.54) ^a^	RR 0.78 (0.41, 1.37)
2	Ji 2018 [37]	Y	OR 1.15 (1.07, 1.24)	OR 1.11 (1.04, 1.20)
3	Kao 2020 [27]	Y	OR 1.03 (0.91, 1.18)	OR 1.01 (0.89, 1.15)
4	Magelssen 2007 [29]	Y	OR 1.02 (0.90, 1.16) ^a^	-
5	Nielsen 2017 [33]	Y	OR 1.16 (0.86, 1.56)	OR 0.99 (0.67, 1.44)
6	Seppanen 2016 [32]	Y	OR 1.17 (0.98, 1.40) ^a^	PR 1.01 (0.83, 1.23)
7	Smaldone 2010 [40]	Y	RR 0.33 (0.08, 1.35)	-
8	Stensheim 2013 [31]	Y	OR 1.17 (0.98, 1.40) ^a^	-
Low birthweight at term				
1	Hartnett 2017 [45]	N	Could not pool	-
2	Hartnett 2018 [41]	N	Could not pool	-
3	Stensheim 2013 [31]	Y	OR 1.28 (0.92, 1.47)	-
Very preterm birth				
1	Anderson 2017b [42]	Y	OR 1.80 (1.44, 2.26) ^a^	PR 2.03 (1.62, 2.55)
2	Hartnett 2017 [45]	N	Could not pool	-
3	Stensheim 2013 [31]	Y	OR 1.22 (0.88, 1.70) ^a^	-
Perinatal death				
1	Magelssen 2007 [29]	Y	OR 0.60 (0.32, 1.12) ^a^	-
2	Smaldone 2010 [40]	Y	RR 3.63 (0.90, 14.7)	-
3	Stensheim 2013 [31]	Y	OR 0.72 (0.51, 1.03) ^a^	-
Neonatal mortality				
1	Farland 2020 [44]	Y	RR 1.55 (0.86, 2.79)	RR 1.30 (0.75, 2.25)
2	Haggar 2014 [36]	Y	OR 1.01 (0.59, 1.75) ^a^	RR 1.03 (0.54, 1.71)
Admission to special/intensive care				
1	Haggar 2014 [36]	Y	OR 1.44 (1.11, 1.86) ^a^	RR 1.44 (1.13, 1.78)
2	Hartnett 2017 [45]	N	Could not pool	-
Sex ratio				
1	Haggar 2014 [36]	Y	OR 1.04 (0.93, 1.16) ^a^	RR 1.05 (0.98, 1.10)
2	Smaldone 2010 [40]	Y	RR 0.92 (0.66, 1.30)	-
Large for gestational age				
1	Kao 2020 [27]	Y	OR 1.03 (0.93, 1.14)	OR 1.03 (0.93, 1.14)
Stillbirth				
1	Kao 2020 [27]	Y	OR 1.05 (0.76, 1.45)	OR 1.01 (0.74, 1.40)
High birthweight				
1	Haggar 2014 [36]	Y	OR 1.25 (1.04, 1.50) ^a^	RR 1.33 (0.99, 1.71)
Very low birthweight				
1	Hartnett 2017 [45]	Y	OR 1.65 (1.34, 2.04) ^a^	-
Neonatal prolonged hospital stay				
1	Farland 2020 [44]	Y	RR 1.19 (1.03, 1.38)	RR 1.16 (1.01, 1.34)
Infectious disease conditions				
1	Farland 2020 [44]	Y	RR 1.12 (0.86, 1.46)	RR 1.04 (0.81, 1.33)
Cardiovascular disease conditions				
1	Farland 2020 [44]	Y	RR 1.09 (0.85, 1.39)	RR 0.90 (0.71, 1.14)
Respiratory conditions				
1	Farland 2020 [44]	Y	RR 1.19 (1.07, 1.33)	RR 1.04 (0.94, 1.14)
Gastrointestinal conditions				
1	Farland 2020 [44]	Y	RR 1.43 (1.22, 1.68)	RR 1.17 (1.02, 1.35)
Neurologic conditions				
1	Farland 2020 [44]	Y	RR 1.03 (0.84, 1.26)	RR 1.06 (0.87, 1.29)
Hematologic conditions				
1	Farland 2020 [44]	Y	RR 1.10 (0.93, 1.30)	RR 0.98 (0.84, 1.14)
**FETAL/NEONATAL-MATERNAL HEALTH OUTCOMES (n = 23 outcomes)**				
During pregnancy (n = 13 outcomes)				
Antepartum hemorrhage				
1	Haggar 2014 [36]	Y	OR 0.91 (0.51, 1.60) ^a^	RR 0.92 (0.59, 1.78)
2	Lee 2016 [28]	Y	OR 0.81 (0.20, 3.27)	OR 1.07 (0.25, 4.55)
Spontaneous abortion				
1	Jacob 2017 [34]	Y	OR 0.59 (0.14, 2.52)	-
2	Lee 2019 [35]	Y	OR 1.03 (0.84, 1.25) ^a^	OR 1.05 (0.86, 1.27)
Post-term pregnancy				
1	Haggar 2014 [36]	Y	OR 0.78 (0.64, 0.95) ^a^	OR 1.04 (0.94, 1.56)
Obstetric hemorrhage				
1	Lee 2019 [35]	Y	OR 0.99 (0.74, 1.33) ^a^	OR 1.00 (0.75, 1.34)
Hydroaminos/Oligo				
1	Lee 2019 [35]	Y	OR 1.11 (0.81, 1.53)	OR 1.15 (0.83, 1.58)
Placental previa				
1	Lee 2016 [28]	Y	OR 1.22 (0.27, 5.52)	OR 1.55 (0.33, 7.25)
Plural birth				
1	Lee 2019 [35]	Y	OR 0.80 (0.50, 1.28) ^a^	OR 0.83 (0.52, 1.33)
Threatened abortion				
1	Haggar 2014 [36]	Y	OR 2.04 (1.49, 2.80) ^a^	RR 2.09 (1.51, 2.74)
Threatened preterm labor				
1	Haggar 2014 [36]	Y	OR 1.31 (0.93, 1.84) ^a^	RR 1.28 (0.88, 1.88)
Medical abortion				
1	Jacob 2017 [34]	Y	OR 1.12 (0.44, 2.83)	-
Unspecified abortion				
1	Jacob 2017 [34]	Y	OR 0.40 (0.21, 0.76)	-
Hemorrhage in early pregnancy without fetal loss				
1	Jacob 2017 [34]	Y	OR 0.53 (0.27, 1.05)	-
Preterm contractions without preterm birth				
1	Jacob 2017 [34]	Y	OR 0.43 (0.22, 0.84)	-
Delivery (n = 9 outcomes)				
Caesarean delivery				
1	Anderson 2017b [42]	Y	OR 1.12 (1.02, 1.22) ^a^	PR 1.08 (1.01, 1.14)
2	Anderson 2018 [39]	Y	OR 1.27 (1.02, 1.59) ^a^	PR 1.14 (1.00, 1.31)
3	Farland 2020 [44]	Y	RR 1.17 (1.11, 1.23)	RR 1.05 (1.00, 1.11)
4	Haggar 2014 [36]	Y	OR 2.95 (2.50, 3.48) ^a^	RR 2.62 (2.22, 3.04)
5	Hartnett 2017 [45]	N	Could not pool	-
6	Hartnett 2018 [41]	N	Could not pool	-
7	Jacob 2017 [34]	Y	OR 0.85 (0.49, 1.49)	-
8	Kao 2020 [27]	Y	OR 1.20 (1.12, 1.28)	OR 1.18 (1.10, 1.27)
9	Smaldone 2010 [40]	Y	RR 1.01 (0.66, 1.53)	-
10	Stensheim 2013 [31]	Y	OR 1.89 (1.74, 2.07) ^a^	-
Premature ruptured membranes				
1	Haggar 2014 [36]	Y	OR 1.05 (0.82, 1.34) ^a^	RR 0.99 (0.83, 1.31)
2	Lee 2019 [35]	Y	OR 0.85 (0.70, 1.03) ^a^	OR 0.83 (0.68, 1.01)
3	Smaldone 2010 [40]	Y	RR 1.19 (0.49, 2.90)	-
Failure to progress				
1	Haggar 2014 [36]	Y	OR 1.50 (0.95, 2.35) ^a^	RR 1.51 (0.97, 2.37)
Fetal malpresentation				
1	Jacob 2017 [34]	Y	OR 0.77 (0.34, 1.75)	-
Preterm labour				
1	Lee 2019 [35]	Y	OR 1.36 (1.09, 1.69) ^a^	OR 1.33 (1.06, 1.65)
Fetal distress				
1	Kao 2020 [27]	Y	OR 1.14 (0.99, 1.31)	OR 1.14 (0.99, 1.31)
Spontaneous delivery				
1	Jacob 2017 [34]	Y	OR 1.96 (1.26, 3.05)	-
Full-term delivery				
1	Lee 2019 [35]	Y	OR 0.78 (0.68, 0.90) ^a^	OR 0.78 (0.68, 0.90)
Successful delivery				
1	Lee 2016 [28]	Y	OR 2.57 (1.69, 3.90)	OR 2.85 (1.83, 4.43)
After delivery (n = 1 outcome)				
Disorders of breast and lactation associated with childbirth				
1	Jacob 2017 [34]	Y	OR 1.77 (0.68, 4.62)	-
**MATERNAL/PATERNAL HEALTH OUTCOMES (n = 2 outcomes)**				
Before pregnancy (n = 1 outcome)				
Fertility treatment				
1	Farland 2020 [44]	Y	OR 2.46 (2.20, 2.75) ^a^	-
2	Haggar 2014 [36]	Y	OR 3.03 (1.02, 4.53) ^a^	RR 1.94 (1.36, 2.69)
3	Magelssen 2007 [29]	Y	OR 10.12 (7.60, 13.57) ^a^	-
4	Stensheim 2011 [30]	Y	OR 2.82 (2.30, 3.45) ^a^	-
5	Stensheim 2013 [31]	Y	OR 2.82 (2.30, 3.45) ^a^	-
6	Velez 2021 [26]	Y	RR 1.30 (1.23, 1.37)	-
After delivery (n = 1 outcome)				
Birth rate				
1	Lee 2019 [35]	Y	HR 0.44 (0.41, 0.47) ^a^	HR 0.41 (0.38, 0.44)
2	Stensheim 2011 [30]	N	Could not pool	-

^a^ Odds ratio calculated from crude numbers. ^b^ Dash: study did not report this measure of association.
